# Endometrial Microbiome and Implantation: From Basic Knowledge to Clinical Medicine

**DOI:** 10.1002/rmb2.70040

**Published:** 2026-03-12

**Authors:** Daiki Hiratsuka, Mitsunori Matsuo, Yasushi Hirota

**Affiliations:** ^1^ Department of Obstetrics and Gynecology, Graduate School of Medicine The University of Tokyo Tokyo Japan

**Keywords:** dysbiosis, endometrium, implantation, Lactobacillus, microbiota

## Abstract

**Background:**

Recurrent implantation failure (RIF) is an infertility condition in which uterine factors remain difficult to diagnose and treat. Recent studies implicate the endometrial microbiome in implantation.

**Methods:**

This clinically oriented narrative review summarizes female reproductive tract microbiota and evidence on endometrial microbiome testing and management in infertility.

**Main Findings (Results):**

Vaginal dysbiosis is linked to adverse reproductive outcomes and provides a reference for interpreting upper‐tract findings. Endometrial microbial signals are detectable by sequencing, but interpretation is challenged by the low‐biomass environment and vulnerability to carry‐over, kitome effects, and contamination. Across ART studies, a *Lactobacillus*‐enriched endometrial profile is more often associated with favorable pregnancy‐related outcomes, whereas non–*Lactobacillus*‐dominant patterns are more frequently reported in implantation failure, although effect sizes and statistical significance vary across cohorts and depend on sampling validity and cutoff definitions. Limited nonrandomized intervention studies suggest that testing‐guided targeted management (typically antibiotics with or without vaginal *Lactobacillus*‐containing probiotics) may benefit selected patients, but protocols are heterogeneous and results remain inconsistent.

**Conclusion:**

Evidence is rapidly evolving, yet observational designs and methodological variability limit causal inference. Future progress will require standardized sampling and contamination controls, outcome‐anchored threshold validation, and pragmatic real‐world evaluations of protocolized test‐and‐treat pathways using clinically meaningful endpoints.

## 1 Introduction

Infertility is a significant social concern affecting couples of reproductive age worldwide, with approximately 17.5% of couples desiring pregnancy experiencing infertility for over 1 year [1]. Infertility treatments typically progress through a stepwise approach, including timed intercourse, intrauterine insemination (IUI), and assisted reproductive technology (ART) [2]. However, a group of patients remains unable to conceive despite undergoing ART. The primary causes of ART failure are embryonic and uterine factors [3]. While advancements in preimplantation genetic testing (PGT) have enabled screening of embryonic abnormalities [4], the diagnosis and treatment of uterine factors remain inadequately established, except for surgical interventions targeting visible lesions, such as endometrial polyps or uterine fibroids [3, 5, 6, 7]. In clinical practice, recurrent implantation failure (RIF) is typically defined as the failure to achieve pregnancy despite transferring at least four good‐quality embryos over three or more cycles in women under 40 years of age [3]. RIF is a form of refractory infertility. While some reports suggest that its incidence is less than 5% [8], others indicate that it may be as high as 20% [3]. Thus, identifying uterine factors contributing to RIF and establishing treatment strategies remain urgent clinical challenges.

The human body harbors microbiomes in various organs, such as the gastrointestinal tract and oral cavity, which play essential roles in maintaining physiological functions; disruptions in these microbiomes are known to be associated with various diseases [9]. In contrast, although the vaginal microbiome is well recognized, the endometrium has traditionally been considered sterile [10]. However, advancements in next‐generation sequencing techniques have revealed the presence of an endometrial microbiome, which is believed to play a critical role in maintaining uterine homeostasis and influencing successful pregnancy outcomes [11, 12, 13]. Therefore, the endometrial microbiome represents a promising avenue for patients with RIF and has emerged as a focus for current research.

Several comprehensive reviews have summarized the emerging evidence linking the endometrial microbiome with implantation and reproductive outcomes [14, 15, 16]. Building on these efforts, the present review is intentionally oriented toward clinical translation in infertility practice, where endometrial microbiome testing is increasingly used despite substantial methodological and interpretive uncertainty. Accordingly, we focus on three practical gaps that are often underemphasized in prior summaries: (i) interpretation of endometrial microbiome testing in a low‐biomass environment, including sampling‐related carry‐over, kitome effects, and contamination control; (ii) the ongoing debate regarding operational definitions of endometrial eubiosis (e.g., *Lactobacillus*‐dominant thresholds) and sensitivity to alternative cut‐offs, including outcome‐based threshold optimization; and (iii) interpretation of the treatment literature at the strategy level—protocolized screening followed by targeted intervention—rather than as definitive support for any single antibiotic or probiotic regimen.

This article is a clinically oriented narrative review of the endometrial microbiome in relation to implantation and reproductive outcomes, with a structured evidence‐synthesis approach. Given substantial heterogeneity in patient populations, sampling strategies, sequencing workflows, and reproductive outcome definitions, a formal systematic review and meta‐analysis was not considered appropriate. Instead, we organized the available evidence using predefined domains (study design, sampling/contamination controls, operational definitions of eubiosis, and reproductive endpoints) and summarise key study characteristics and outcome signals in structured tables to support transparent interpretation in clinical practice.

## 2 Microbiome

### 2.1 Microbiome and Microbiota

The microbiome refers to the collective community of microorganisms—including bacteria, fungi, viruses—and their genetic material that inhabit a defined environment [17]. In humans, microbiomes are found across multiple sites such as the gut, nose, oral cavity, skin, and reproductive tract [17]. The organisms themselves are termed microbiota. Importantly, the microbiome concept extends beyond taxonomic composition to include microbial interactions, functions, and metabolites (e.g., proteins, peptides, lipids, polysaccharides, nucleic acids, signaling molecules, organic molecules, toxins) as well as environmental conditions that shape host–microbe crosstalk and homeostasis [18]. For example, the gut microbiome is the most abundant and is influenced by diverse factors, including age, genetics, ethnicity, antibiotics, prebiotics, probiotics, pollution, diet, smoking, alcohol consumption, hygiene, stress, and chemotherapy [19]. Microbial communities also form networks across organs such as the brain, heart, liver, and reproductive tract, underscoring their systemic relevance to human physiology [19].

### 2.2 Dysbiosis and Diseases

Eubiosis and dysbiosis represent two contrasting states of the microbiome. Eubiosis denotes a balanced microbial community in which beneficial bacteria dominate and support homeostasis and immune function [20]. Dysbiosis is characterized by a loss of beneficial taxa, overgrowth of pathogenic species, and reduced microbial diversity [21], potentially impairing protective functions, compromising immunity, increasing toxin production, and disrupting mucosal integrity. For example, gut dysbiosis has been linked to inflammatory bowel disease (IBD), metabolic disorders, autoimmune conditions, polycystic ovary syndrome (PCOS), and infertility [22, 23]. The gut–reproductive axis further suggests that gut dysbiosis may influence gynecological conditions such as endometriosis and PCOS via systemic inflammation and hormonal imbalance [24]. Overall, dysbiosis is increasingly recognized as a contributor to disease pathogenesis beyond the gut.

### 2.3 Microbiome Analysis Techniques

Advances in microbiome analysis have enabled comprehensive profiling of complex ecosystems, including low‐biomass environments such as the endometrium. Traditional culture‐based approaches, including culturomics, use diverse media and incubation conditions to maximize recovery of viable organisms, but often underestimate community diversity because non‐culturable or slow‐growing species may not be detected [25, 26]. In contrast, 16S rRNA gene sequencing is an amplicon‐based method that targets conserved and variable regions (V1–V9) to generate bacterial taxonomic profiles after DNA extraction, PCR amplification, and next‐generation sequencing, and it is commonly applied to low‐biomass samples such as endometrial tissue [27]. Whole‐genome shotgun metagenomic sequencing provides untargeted, higher‐resolution characterization of all genetic material in a sample, enabling profiling of bacteria, viruses, fungi, and archaea, and improving taxonomic accuracy relative to 16S approaches [28]. Beyond DNA‐based profiling, metatranscriptomics, metaproteomics, and metabolomics aim to capture microbial activity and functional outputs. Metabolomics, for example, can quantify microbially derived metabolites such as short‐chain fatty acids (SCFAs), bile acids, and amino acids [28], which are increasingly recognized as mediators of inflammation, immune regulation, and potentially endometrial receptivity. Collectively, these approaches are also enabling strain‐level characterization, including gene functions, antibiotic resistance, and virulence factors, thereby enhancing clinical relevance.

### 2.4 Treatments for Dysbiosis

Because dysbiosis is implicated in diverse diseases, strategies to modulate the microbiome have gained attention, including diet, antibiotics, prebiotics, probiotics, postbiotics, phage therapy, microbiota transplantation, and live biotherapeutic products (LBPs). Diet is a fundamental determinant of microbiome composition, as it provides substrates that preferentially support beneficial or pathogenic taxa [29]. In particular, high‐fiber, plant‐based dietary patterns promote intestinal fermentation and enrichment of SCFA‐producing microbiota, which can strengthen epithelial barrier function and attenuate systemic inflammation [30]. Antibiotics remain widely used to treat bacterial overgrowth in dysbiosis‐related conditions, including chronic endometritis [31], yet they may disrupt commensals, induce secondary dysbiosis, and contribute to antimicrobial resistance, underscoring the need to avoid unnecessary or repeated exposure [32].

Prebiotics are nondigestible compounds that selectively stimulate beneficial organisms; for example, lactoferrin has been reported to influence *Lactobacillus* dominance in the vagina [33]. Probiotics are live microorganisms that confer health benefits when administered in adequate amounts [34], and *Lactobacillus*‐based preparations are frequently used in the female reproductive tract, where they may act via competitive exclusion and antimicrobial production such as bacteriocins [35]. Postbiotics—microbially produced metabolites including SCFAs, bacteriocins, and enzymes—have attracted interest because they can exert anti‐inflammatory, antimicrobial, and immunomodulatory effects without administering live organisms [36].

Phage therapy offers a targeted approach by using bacteriophages to selectively eliminate pathogenic bacteria while preserving beneficial taxa [37]. Microbiota transplantation transfers donor communities to restore eubiosis; fecal microbiota transplantation is the best‐studied example, and vaginal microbiota transplantation has been explored as an analogous strategy within the reproductive tract [38]. Finally, LBPs represent microbiome‐based therapeutics composed of defined strains or consortia selected for specific beneficial properties, conceptually distinct from traditional probiotics [38].

## 3 Microbiome in the Female Reproductive Tract

### 3.1 Microbiome in the Female Reproductive Tract

**FIGURE 1:**
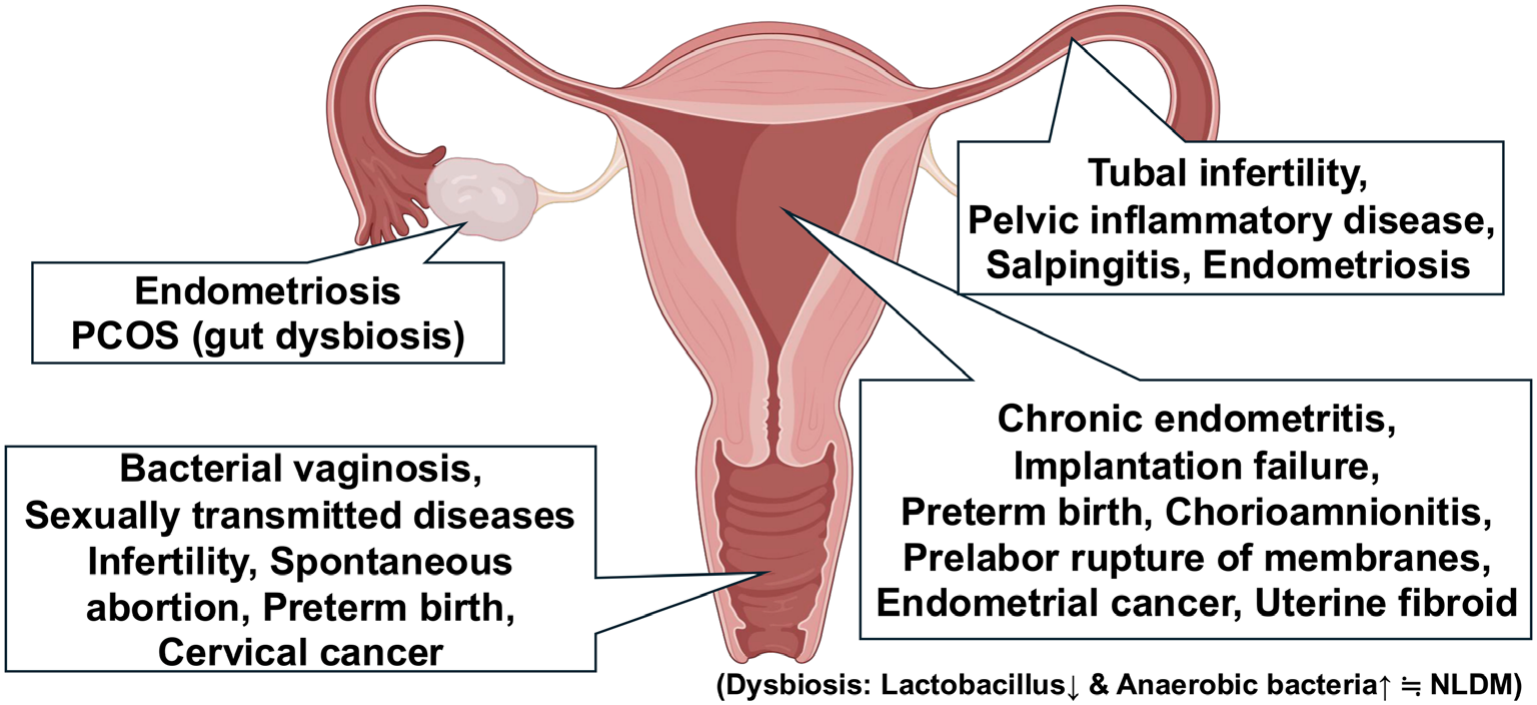
Abnormal microbiome and diseases in female reproductive tract.

The vagina has long been recognized to harbor a microbiome, whereas the endometrium was traditionally considered sterile. With advances in next‐generation sequencing (NGS), bacterial DNA has been detected not only in the vagina but also in the cervix, endometrium, oviducts, and ovary, challenging the concept of sterility in the upper reproductive tract [10, 39, 40]. In healthy women, *Lactobacillus* species commonly dominate microbial communities across the lower genital tract, while dysbiosis has been associated with a range of gynecologic and obstetric conditions (Figure 1) [41, 42, 43, 44, 45, 46]. Because the vaginal microbiome is a well‐established, anatomically adjacent ecosystem with robust clinical literature, it provides a practical reference framework when interpreting endometrial microbiome data—particularly for understanding shared taxa, distinguishing site‐specific profiles, and contextualizing methodological concerns such as potential carry‐over and low‐biomass signal validity. Accordingly, we briefly summarize key concepts of the vaginal microbiome as a comparator before focusing on endometrial microbiota.

### 3.2 Vaginal Microbiome

The healthy vaginal microbiota is primarily dominated by *Lactobacillus* species, which maintain an acidic environment (pH 3.5–4.5) [47, 48], contain approximately 10^10^–10^11^ bacteria, and exhibit less diversity compared to other human microbiotas [49, 50, 51]. Vaginal communities are often categorized into Community State Types (CSTs) based on dominant taxa [52].

- CST‐I: *Lactobacillus crispatus* –dominant; stable, healthy vaginal environment with the strongest protection against infection.
- CST‐II: *Lactobacillus gasseri* –dominant; generally healthy but less stable than CST‐I.
- CST‐III: *Lactobacillus iners* –dominant; still considered healthy but relatively unstable and more likely to transition to dysbiosis.
- CST‐IV: *Lactobacillus*‐depleted with anaerobic overgrowth (e.g., *Gardnerella vaginalis, Prevotella, Atopobium vaginae*); associated with BV and inflammation.
- CST‐V: *Lactobacillus jensenii* –dominant; moderately protective and relatively rare.

### 3.3 Functions of Vaginal Microbiome

*Lactobacilli* support vaginal health by producing lactic acid and antimicrobial factors (including bacteriocins), thereby suppressing pathogen overgrowth and contributing to mucosal barrier integrity and immune homeostasis [50, 51, 53]. The vaginal microbiome is dynamic and influenced by hormonal states (menstrual cycle, hormonal therapy, pregnancy) that alter glycogen availability and thereby favor *Lactobacillus* dominance; pregnancy is often associated with increased stability and reduced diversity [54, 55]. Sexual activity and semen exposure can transiently raise vaginal pH, potentially reducing *Lactobacillus* dominance and promoting anaerobic growth [56, 57, 58]. These characteristics suggest that sampling timing and clinical context can meaningfully affect microbiome assessments.

### 3.4 Vaginal Dysbiosis and Diseases

Vaginal dysbiosis is typically defined by reduced *Lactobacillus* abundance with expansion of anaerobic/pathogenic taxa, leading to higher pH, weakened colonization resistance, and inflammation [39]. Bacterial vaginosis (BV) is the most recognized dysbiotic state and has been linked to adverse reproductive outcomes. A meta‐analysis reported increased early pregnancy loss and reduced clinical pregnancy rates in IVF among women with abnormal vaginal microbiota [59]. Vaginal dysbiosis has also been associated with preterm birth risk, particularly when *Lactobacillus crispatus* dominance is reduced [54], as well as pregnancy‐related infections and complications such as chorioamnionitis, postpartum endometritis, and premature rupture of membranes (PROM) [60, 61]. In infertility populations, BV and/or abnormal vaginal microbiota appear more prevalent than in pregnant controls [62]. Emerging evidence also suggests links between lower genital tract dysbiosis and conditions such as endometriosis and uterine fibroids [44, 45], although causality remains uncertain.

### 3.5 Treatments for Vaginal Dysbiosis

Current interventions aim to restore vaginal eubiosis and reduce recurrence, but treatment protocols remain heterogeneous. Antibiotics (metronidazole or clindamycin) are standard for BV and achieve high initial cure rates; however, recurrence is common, and repeated exposure may disrupt beneficial *Lactobacillus* and contribute to antimicrobial resistance [63, 64, 65, 66, 67]. Prebiotics such as lactoferrin and oligosaccharides have been explored to support *Lactobacillus* dominance, including immunomodulatory effects in the vaginal mucosa [68, 69, 70, 71, 72, 73, 74, 75, 76, 77]. Probiotics, particularly *Lactobacillus*‐based formulations administered orally or intravaginally, have been investigated with mixed results across populations and protocols; efficacy may depend on timing, strain selection, route, and baseline dysbiosis status [78, 79, 80, 81, 82, 83, 84, 85]. Postbiotics (e.g., *Lactobacillus*‐derived metabolites) may provide antimicrobial and symptom‐improving effects without administering live organisms [86, 87]. Vaginal microbiota transplantation (VMT) is an emerging strategy with early evidence in recurrent or refractory BV, but clinical data remain limited, including in infertility settings [88, 89]. Overall, while antibiotics remain the mainstay, adjunct microbiome‐targeted strategies are increasingly studied to improve durability of eubiosis and reproductive outcomes.

## 4 Microbiome in Endometrium

### 4.1 The Existence of Endometrial Microbiome

As mentioned in Section 3.1, *the microbiome in the female reproductive tract*, the human uterus was long believed to be a sterile environment. This belief stemmed from the limitations of traditional microbiological techniques, which relied on aerobic and anaerobic culture methods incapable of detecting low‐abundance or fragile microorganisms. Consequently, the presence of bacteria in the upper genital tract was generally regarded as pathological, typically indicating pelvic inflammatory disease or an ascending infection from the lower tract. This paradigm has been challenged over the past decade by the advent of next‐generation sequencing (NGS) technologies, particularly 16S rRNA gene sequencing. These culture‐independent methods allow for the detection and taxonomic classification of bacterial DNA in low‐biomass tissues. In 2016, Moreno et al. reported that the endometrium harbors a unique microbiome distinct from that of the vagina. Using paired endometrial and vaginal samples, they demonstrated that the microbial profiles at the two sites differed significantly. However, this emerging field of research remains controversial. Some researchers have argued that the detected microbial DNA might originate from the lower genital tract or environmental contamination. This concern has been addressed in several studies. For example, true microbial signals were distinguished from background noise using double‐sheathed catheter sampling, including air and saline controls, and abundance thresholds derived from a fertile reference population were used [90]. Paired sampling of the cervix, vagina, and endometrium confirmed distinct microbial compositions at each site [91]. Moreover, endometrial microbiota have also been detected in samples obtained from hysterectomies [92]. There is now substantial evidence that the endometrium harbors its own microbiota and that its composition may critically influence implantation and pregnancy outcomes.

### 4.2 The Composition of a Healthy Endometrial Microbiota

A healthy endometrial microbiome in women of reproductive age is generally defined by a predominance of *Lactobacillus* species. This *lactobacillus* dominant microbiota (LDM) is considered a hallmark of eubiosis and is associated with enhanced endometrial receptivity, reduced inflammation, and improved implantation outcomes, whereas a non‐*Lactobacillus*‐dominant state is termed NLDM, indicating endometrial dysbiosis [11]. The dominant species identified in the endometrium were *Lactobacillus crispatus* , *L. iners* , *L. gasseri* , and *L. jensenii* [93]. Among these, *L. crispatus* is most consistently associated with optimal reproductive outcomes, whereas *L. iners* exhibits dual properties depending on the strain‐specific genomic profiles [93]. In addition to *Lactobacillus*, the other bacterial species detected included *Bifidobacterium*, *Propionibacterium*, *Gardnerella*, *Streptococcus*, and *Veillonella* [11]. Studies comparing endometrial and vaginal microbiota have shown that, although the two share certain taxa, their relative abundances and characteristics differ markedly [94, 95]. Notably, the endometrial microbiota is of much lower biomass than the vaginal microbiota; the quantity of endometrial bacteria is approximately 1/100–1/10000 of the vagina microbiota, and its composition remains relatively diverse and stable even during endometrial receptivity acquisition [11]. Therefore, a healthy endometrial microbiota is typically *Lactobacillus*‐rich, immunotolerant, and hormonally synchronized. The composition of the endometrial microbiota is governed by local tissue‐specific factors, and dysbiosis cannot be inferred from vaginal analysis alone.

### 4.3 Mechanisms of Lactobacillus‐Dominated Microbiota in Endometrial Health

The presence of LDM in the endometrium is increasingly recognized as a key component of uterine health and fertility. Lactobacilli, particularly *L. crispatus* , *L. gasseri* , and *L. jensenii* , contribute to a favorable microenvironment through multiple synergistic mechanisms, including immunomodulation, pathogen exclusion, and biochemical support for implantation [48, 93, 96, 97].

#### 4.3.1 Acidification and Antimicrobial Activity

One of the primary functions of *Lactobacillus* is the production of lactic acid, which maintains a low pH in the vaginal tract and possibly in the upper reproductive tract. Although the uterine environment is not as acidic as the vagina, localized acidification may contribute to antimicrobial activity, limiting the proliferation of pathogenic anaerobes, such as *Gardnerella*, *Atopobium*, and *Streptococcus* [11, 90]. In addition to lactic acid, *lactobacilli* produce hydrogen peroxide and bacteriocins, which suppress bacterial overgrowth and maintain microbial balance [98].

#### 4.3.2 Immune Regulation

*Lactobacilli* exert immunomodulatory effects by influencing the expression of pattern recognition receptors on endometrial epithelial and immune cells, such as TLR2, TLR4, and TLR6 [99, 100]. These interactions can downregulate NF‐κB signaling and reduce the expression of proinflammatory cytokines (e.g., IL‐6, IL‐8, TNF‐α), thereby preventing a shift toward a Th1‐dominant or inflammatory milieu [99, 101, 102, 103]. The balance of immune tolerance is particularly critical during the implantation window, where excessive inflammation may impair decidualization and embryo acceptance [104].

#### 4.3.3 Barrier and Decidual Support

*Lactobacilli* may support the integrity of the endometrial barrier by enhancing tight junctions and mucin production. *L. crispatus* promotes the invasion of extravillous trophoblasts in vitro, suggesting that certain *Lactobacillus* strains directly enhance implantation competence [105]. Furthermore, *Lactobacillus* colonization has been associated with increased expression of matrix metalloproteinases (MMPs), which facilitate controlled tissue remodeling and embryo invasion [105].

#### 4.3.4 Suppression of Biofilm and Pathogen Colonization

A healthy *Lactobacillus* community prevents colonization by biofilm‐forming pathogens such as *Gardnerella vaginalis* or *Escherichia coli* . This phenomenon is particularly relevant in chronic endometritis and RIF, in which biofilm persistence may shield pathogens from antibiotic eradication. *Lactobacilli* competitively exclude these organisms by occupying adhesion sites and secreting antiadhesive compounds [106].

#### 4.3.5 Species‐Specific Effects

However, not all *Lactobacillus* species confer the same level of protection. Although *L. crispatus* is considered a robust colonizer with potent immunoprotective effects, it is also associated with transitional or dysbiotic states. Its genome encodes cytolytic proteins such as inerolysin, which may contribute to epithelial damage under certain conditions. The *L. iners* ‐dominant microbiota was associated with significantly lower implantation and pregnancy rates than *L. crispatus* ‐dominant communities in patients undergoing IVF [93].

In summary, *Lactobacillus* serves as a guardian of endometrial eubiosis through multifaceted mechanisms. Their metabolic, immune, and ecological contributions create a receptive uterus, highlighting the importance of preserving or restoring Lactobacillus dominance in reproductive medicine.

### 4.4 Endometrial Dysbiosis and Implantation Failure

Endometrial dysbiosis, characterized by NLDM, has emerged as a significant contributing factor to implantation failure. Although associations with miscarriage and recurrent pregnancy loss have also been suggested [107, 108], the focus here is on organizing the evidence related to implantation failure, with key methodological limitations discussed in Section 4.7.4.

Across studies in IVF/ART populations (Table 1), a *Lactobacillus*‐enriched endometrial profile tends to be associated with higher pregnancy or live birth outcomes, whereas reduced *Lactobacillus* dominance and/or enrichment of specific taxa is more frequently reported among patients with implantation failure [11, 91, 109, 110, 111, 112]. However, the magnitude and statistical significance of these associations are inconsistent, likely reflecting heterogeneity in study design, patient selection (e.g., RIF vs. broader IVF cohorts), sampling/contamination control, sequencing pipelines, outcome definitions, and the operational definition of “eubiosis” (Section 4.7).

**TABLE 1:** Observational studies of pregnancy outcomes in relation to *Lactobacillus*‐dominant or non–*Lactobacillus*‐dominant endometrial microbiota.

Study	Population	LDM definition	Outcomes	Target of 16S rRNA sequencing	Hormonal cycle
Moreno et al. (2016)	IVF patients (*n* = 35)	LDM ≥ 90% vs. NLDM < 90%	Implantation rate: 60.7% vs. 23.1% Pregnancy rate: 70.6% vs. 33.3% Ongoing pregnancy: 58.8% vs. 13.3% Live birth: 58.8% vs. 6.7%	V3‐V5	Secretory
Miyagi et al. (2023)	IVF patients (*n* = 33)	LDM ≥ 46% and PB < 18.7% vs. NLDM < 46% and PB ≥ 18.7% (based on ROC curve)	Pregnancy rate: 77.3% vs. 16.7%	V1‐V2	Proliferative
Moreno et al. (2022)	IVF patients (*n* = 342)	Not Applicable	Lactobacillus was enriched in patients with live birth outcomes Dysbiotic endometrial microbiota was observed in patients with unsuccessful outcomes	V2‐4 and V6‐9	Secretory
Hashimoto et al. (2019)	IVF patients (*n* = 99)	Lactobacillus + Bifidobacterium ≥ 80% vs. Lactobacillus + Bifidobacterium < 80%	Implantation rate: 52.9% vs. 53.1% Pregnancy rate: 52.9% vs. 54.8%	V4	Secretory
Bui et al. (2023)	IVF patients after the failure of the first ET (*n* = 92)	Not Applicable	*Lactobacillus crispatus* 45.8% in patients with live birth vs. *Lactobacillus crispatus* 25.4% in patients without live birth	V1‐V2	Secretory
Diaz‐Martínez et al. (2021)	IVF patients (*n* = 48)	Not Applicable	Lactobacillus abundance was higher in patients with pregnancy than those without pregnancy	V3‐V4	Secretory
Vomstein et al. (2022)	RIF/RM patients (*n* = 50)	Not Applicable	Lactobacillus abundance was lower in RIF/RM patients than in controls	V3‐V4	Secretory
Kyono et al. (2018)	IVF patients (*n* = 92)	① LDM ≥ 90% vs. NLDM < 90% ② LDM ≥ 80% vs. NLDM < 80%	① Pregnancy rate: 58.9% vs. 47.2% ② Pregnancy rate: 61.3% vs. 40.0%	Not applicable	Secretory

Abbreviations: ET, embryo transfer; IVF, in vitro fertilization; LDM, Lactobacillus‐dominant microbiota; NLDM, non–Lactobacillus‐dominant microbiota; RIF, recurrent implantation failure; RM, recurrent miscarriage.

In the landmark prospective study by Moreno et al., higher pregnancy rates were observed among patients with LDM compared with NLDM, and miscarriage rates were higher in NLDM [11]. In a subsequent report, specific community patterns and enrichment of taxa such as *Atopobium*, *Gardnerella*, *Streptococcus*, and others were associated with poorer reproductive outcomes [109]. Other cohorts have similarly reported that higher *Lactobacillus* abundance—particularly enrichment of *Lactobacillus crispatus* —is associated with favorable outcomes, including live birth [91, 110]. Additional prospective data have suggested differences in alpha diversity and/or *Lactobacillus* abundance between those who conceive and those who do not, although not all metrics reach statistical significance across studies [111]. In cycle‐based analyzes, uterine microbiota plasticity across the menstrual cycle may further contribute to between‐study variability [112]. However, some studies report null associations between LDM/NLDM categories and IVF outcomes [113]. In at least one report, statistical separation was observed when using an alternative threshold (e.g., 80% rather than 90%) to define *Lactobacillus* dominance, highlighting sensitivity to cutoff choice [114]. These inconsistencies underscore that NLDM may be best interpreted as a probabilistic risk marker rather than a deterministic cause of implantation failure (Section 4.7.4).

Taken together, current evidence supports an association between NLDM and poorer reproductive outcomes in subsets of IVF/RIF populations, but heterogeneity and observational designs limit causal inference. Accordingly, NLDM is most appropriately viewed as a potentially relevant prognostic feature within a broader uterine‐factor assessment, with careful attention to sampling validity and definition thresholds (Section 4.7).

### 4.5 Treatments for Endometrial Dysbiosis

Interventions aimed at modulating endometrial microbial profiles have been explored in infertility practice, most commonly using antibiotics with or without *Lactobacillus*‐containing probiotics. Although both oral and vaginal routes have been used, vaginal administration may be more effective for modulating genital tract microbial communities; accordingly, vaginal probiotic strategies have increasingly become the predominant approach in this setting [115]. Importantly, protocols vary widely across studies, and the strongest signal in the current literature is best interpreted at the strategy level—endometrial microbiome testing followed by targeted intervention in selected patients—rather than as evidence supporting any single standardized regimen (Section 4.7.4).

Evidence directly examining whether ART patients with NLDM can be treated and subsequently “converted” to an LDM profile confirmed by repeat endometrial microbiome testing remains limited. Only a small number of clinical reports/pilot studies have explicitly incorporated a test–treat–retest approach, most commonly using antibiotics combined with vaginal *Lactobacillus* formulations and documenting shifts toward *Lactobacillus*‐enriched profiles after intervention [114, 115, 116] (Table 2). Collectively, these data suggest that, in at least some patients, endometrial microbial profiles may be modifiable; however, the number of studies and sample sizes remains insufficient to define expected conversion rates, durability of response, or generalizability across ART settings.

**TABLE 2:** Observational studies of *Lactobacillus*‐containing probiotics for the treatment of non–*Lactobacillus*‐dominant endometrium.

Study	Population	*Lactobacillus*‐containing Probiotics	Duration of probiotic treatment	Co‐interventions	Treatment outcomes
Kyono et al. (2018)	IVF patients (*n* = 9)	*L. rhamnosus* , *L. reuteri*	30 days	Antibiotics, Lactoferrin	100% (9/9) patients with NLDM converted to LDM
Kadogami et al. (2020)	RIF (*n* = 28)	*L. fermentum L. plantarum L. gasseri*	7 days	Antibiotcs	79% (22/28) patients with NLDM converted to LDM
Iwami et al. (2023)	RIF (*n* = 30)	Type 1 *L. fermentum L. plantarum L. gasseri* Type 2 *L. rhamnosus L. reuteri*	7–10 days	Antibiotcs	77% (23/30) patients with NLDM converted to LDM

*Note:* LDM was defined as Lactobacillus abundance ≥ 90%.

Abbreviations: IVF, in vitro fertilization; LDM, Lactobacillus‐dominant microbiota; NLDM, non–Lactobacillus‐dominant microbiota; RIF, recurrent implantation failure.

Reports suggesting improved pregnancy‐related outcomes specifically in programs that identified NLDM and then implemented targeted interventions are currently few. Prospective cohort evidence indicates improved outcomes in IVF/RIF populations when sequencing‐based assessment is coupled with selective treatment of dysbiosis [116]. Multicenter prospective data further suggest that personalizing management of microbial imbalance may shorten time to pregnancy [12]. In addition, our recent study in RIF comparing diagnostic tests for CE and dysbiosis suggested that combining assessments and addressing dysbiosis in clinical practice may be associated with improved pregnancy outcomes [13]. Nonetheless, the total number of such studies remains small, and their designs are largely nonrandomized.

In the more established vaginal microbiome field, intravaginal *Lactobacillus* supplementation for vaginal dysbiosis has shown mixed effects on reproductive outcomes, with some trials reporting benefit and others reporting no improvement [117]. This heterogeneity cautions against assuming that vaginal probiotic effects translate directly to the endometrium. Accordingly, whether and to what extent vaginal *Lactobacillus* supplementation improves reproductive outcomes specifically through correction of endometrial dysbiosis remains uncertain, and additional well‐designed studies focusing on endometrial status and clinically meaningful endpoints are needed.

Several studies have evaluated vaginal *Lactobacillus* supplementation prior to embryo transfer without restricting enrollment to NLDM patients and without confirming endometrial microbiome status after intervention. Across these studies, results are inconsistent, with a larger proportion of studies reporting improved pregnancy‐related outcomes, although several studies have found no measurable benefit [83, 118, 119, 120, 121, 122]. Such heterogeneity may reflect differences in baseline microbiota status, embryo‐related factors, co‐interventions, endpoints, probiotic compositions, and study design.

Therefore, available evidence most consistently supports a pragmatic clinical concept: endometrial microbiome testing–guided, protocolized screening followed by targeted intervention may be beneficial for selected patients, while optimal thresholds, candidate taxa, and standardized regimens remain uncertain and require further validation using clinically meaningful endpoints.

### 4.6 Chronic Endometritis and Endometrial Dysbiosis

Chronic endometritis (CE) is also associated with endometrial dysbiosis. Since CE and endometrial dysbiosis share common elements, such as altered microbiota composition and antibiotic treatment strategies, understanding both conditions in an integrated manner is clinically important. Conceptually, CE and endometrial dysbiosis are related but distinct entities. CE is a clinicopathological diagnosis of persistent endometrial inflammation, whereas endometrial dysbiosis is a microbiome‐based construct reflecting an altered community composition (e.g., reduced *Lactobacillus* dominance and/or enrichment of specific taxa) as assessed by sequencing‐based assays. Therefore, overlap is common, but the two conditions are not interchangeable.

#### 4.6.1 Chronic Endometritis

Endometritis can be classified into two types: acute endometritis, which presents with symptoms such as fever, lower abdominal pain, and abnormal vaginal discharge, and CE, which is characterized by persistent inflammation in the absence of overt symptoms. CE is observed in 30%–57% of patients with RIF or recurrent pregnancy loss while as low as ~0.2% in general gynecologic populations, and it is recognized as one of the uterine factors contributing to infertility [123, 124, 125]. CE is diagnosed by detecting CD138‐positive plasma cells in endometrial tissue or by hysteroscopic findings. CD138 is identified by immunohistochemistry; however, its diagnostic criteria have not been standardized [126, 127]. On hysteroscopy, the presence of erythema, hyperemia, edema, or micropolyps is suggestive of CE, and this method is commonly used as a supplemental tool, along with the endometrial CD138 test [128].

Antibiotic therapy is the mainstay of treatment, and doxycycline for 2 weeks is currently considered the first‐line treatment option. This regimen has been reported to achieve a cure in approximately 88.9% of patients with CE [129]. There have been reports indicating that in patients undergoing embryo transfer after antibiotic treatment for CE, live birth rates are significantly higher than those in patients without CE at baseline. Furthermore, patients with implantation failure who were cured of CE after antibiotic therapy had significantly higher pregnancy and live birth rates than those in whom CE persisted. These findings establish that antibiotic treatment for CE is an important therapeutic approach for patients with implantation failure [124, 125, 127, 130].

However, subsequent studies have reported that antibiotic treatment for CE did not lead to an improvement in pregnancy rates, and that miscarriage rates remained higher than those in patients without a history of CE, even after treatment [129, 131].

#### 4.6.2 Chronic Endometritis and Endometrial Dysbiosis

Patients with implantation failure and CE were highly likely to develop endometrial dysbiosis. One study reported that the median endometrial Lactobacillus abundance was 80.7% in patients without CE, whereas it was only 1.9% in those with CE, suggesting a CE‐specific microbial composition [90, 132]. Another study reported that the endometrial microbiome of CE patients includes *Ureaplasma urealyticum* , *Mycoplasma hominis* , *Escherichia coli* , *Enterococcus faecalis* , *Streptococcus agalactiae* , *Gardnerella vaginalis* , and *Atopobium* species [128]. CE, however, can also exist without endometrial dysbiosis. Previous studies have reported that patients with endometriosis or adenomyosis tend to have a higher prevalence of CE, whereas childbirth is associated with a reduced frequency of CE [133, 134]. Therefore, many fertility institutions now perform a combination of diagnostic evaluations—including hysteroscopy, endometrial CD138 testing, and endometrial microbiota testing—when assessing CE and endometrial dysbiosis [126].

Although a standardized approach regarding which tests to perform, how to interpret them, and how to treat findings to optimize fertility outcomes has not yet been established, recent reports suggest that identifying and treating endometrial dysbiosis among patients who undergo all three tests results in the most favorable pregnancy outcomes [13]. Further evidence is required to validate these findings and establish clear clinical guidelines.

Importantly, discordant patterns can occur. CE may be present despite an apparently *Lactobacillus*‐dominant profile, reflecting that inflammation can persist even when sequencing suggests “eubiosis,” or when tissue‐associated inflammation is not captured by luminal sampling. Conversely, endometrial dysbiosis can be detected in the absence of CE on histology, indicating that compositional shifts do not always translate into plasma cell infiltration at the time of biopsy. These distinctions have practical implications: microbiome results should not be used as a surrogate for CE, and negative CD138 findings do not exclude clinically relevant dysbiosis. When evaluating uterine factors in infertility and recurrent implantation failure, CE and dysbiosis should be considered complementary assessments rather than mutually substitutive diagnoses.

### 4.7 Current Challenges in Endometrial Microbiome Research

Despite growing interest in the role of the endometrial microbiota in implantation and infertility, several methodological challenges limit its routine clinical application. These challenges primarily relate to heterogeneity in the definition of LDM, sampling strategies, contamination, and the intrinsic low‐biomass nature of the endometrial environment, as well as limitations in causal inference and the heterogeneity of interventions across studies.

#### 4.7.1 Heterogeneity in the Definition of Lactobacillus‐Dominant Microbiota

LDM is widely regarded as a marker of endometrial eubiosis and has been associated with enhanced endometrial receptivity, reduced inflammation, and improved implantation outcomes. However, there is no universally accepted cutoff to define LDM. Most early and influential studies defined LDM as ≥ 90% relative abundance of *Lactobacillus* species, a threshold that has been adopted in many subsequent clinical studies [11]. Nevertheless, alternative cutoffs have been proposed. Some investigators have used ≥ 80% *Lactobacillus* abundance to define eubiosis, arguing that this lower threshold may provide greater robustness in low‐biomass samples where relative abundance estimates are sensitive to technical noise [95, 135]. Others have further expanded the definition to include *Bifidobacterium* as a potentially beneficial taxon, defining eubiosis as ≥ 80% *Lactobacillus* plus *Bifidobacterium* [113]. This variability in definitions complicates cross‐study comparisons and may partially explain inconsistent associations between endometrial microbiota status and reproductive outcomes. Furthermore, one IVF cohort derived an outcome‐based cutoff for endometrial *Lactobacillus* abundance using receiver operating characteristic (ROC) analysis (46%) and reported significant differences in pregnancy outcomes across the resulting groups [91], illustrating both the potential utility and cohort‐specificity of threshold optimization. At present, although the definition as ≥ 90% relative abundance of *Lactobacillus* species is the most frequently used, there is insufficient evidence to recommend a single optimal cutoff, underscoring the need for standardized definitions validated against clinically meaningful endpoints such as implantation and live birth. Future studies should externally validate outcome‐based thresholds in independent cohorts, report sensitivity analyzes across plausible cutoffs (e.g., 80%, 90%, and cohort‐derived values), and complement threshold‐based classification with models that treat *Lactobacillus* abundance as a continuous variable—particularly in low‐biomass settings where estimates may be sensitive to contamination and batch effects.

#### 4.7.2 Sampling Methods and Their Limitations

Sampling methodology represents another major source of variability. Because endometrial samples are typically obtained via transcervical routes, they are inherently vulnerable to contamination from the cervix and vagina, which harbor orders of magnitude higher bacterial biomass [136]. Common approaches include aspiration of endometrial fluid, endometrial biopsy using devices such as Pipelle, and analysis of embryo transfer catheter tips. Each method has advantages and limitations. Endometrial fluid aspiration is minimally invasive and easily implemented in clinical practice but is particularly susceptible to carry‐over contamination [136]. Biopsy yields greater tissue mass and potentially higher microbial signal but still requires passage through the cervix and may induce local inflammation or bleeding that could influence microbial profiles. The use of double‐sheathed or double‐lumen embryo transfer catheters has been proposed as a contamination‐reducing strategy, as the catheter tip is exposed only within the uterine cavity [137]. Although this approach may lower the risk of cervical and vaginal carry‐over, it does not fully eliminate contamination and may preferentially sample luminal rather than tissue‐associated microbiota. Importantly, no sampling method can be considered contamination‐free, and differences in sampling approaches across studies further hinder reproducibility.

#### 4.7.3 Contamination, Kitome, and the Low‐Biomass Problem

The endometrium is a quintessential low‐biomass environment, making microbiome analyzes highly susceptible to contamination not only during sampling but also during DNA extraction, library preparation, and sequencing [136, 138, 139]. Reagent‐ and laboratory‐derived bacterial DNA (“kitome”) can dominate sequencing results when true microbial biomass is low, leading to spurious detection of taxa that do not reflect in vivo biology. This issue has been extensively documented in low‐biomass microbiome research and is particularly relevant to endometrial studies. Consequently, rigorous contamination control is essential. Best practices include the use of multiple negative controls at each experimental step (sampling blanks, extraction blanks, and PCR blanks), incorporation of mock community positive controls, and transparent reporting of contamination assessment [140]. Without these measures, it is difficult to distinguish true endometrial signals from background noise. In addition to experimental controls, statistical approaches can support contaminant identification and removal [141], although computational filtering cannot substitute for appropriate study design and laboratory controls.

#### 4.7.4 Limits of Causal Inference and Heterogeneity of Interventions

Most studies evaluating endometrial microbiota status and reproductive outcomes are observational, retrospective, or based on before–after comparisons, which inherently limits causal inference between endometrial dysbiosis and implantation failure. Confounding by indication and co‐interventions are common: patients identified as “non‐LDM” frequently receive additional treatments (e.g., antibiotics, probiotics, or other adjunctive fertility interventions), and embryo‐related factors may not be fully controlled in real‐world settings. RCTs are particularly challenging in this field for both ethical and practical reasons. First, randomizing patients with suspected dysbiosis to “no intervention” or delaying embryo transfer solely for study purposes may be unacceptable in infertility care, where time‐to‐pregnancy is a clinically meaningful outcome. Second, repeated transcervical sampling to confirm microbiome changes can impose additional invasiveness and potential risks (e.g., discomfort, bleeding, or infection), and may not be feasible in routine practice. Third, the interventions themselves are heterogeneous and not standardized, making it difficult to define a single, universally applicable RCT protocol. Indeed, treatment strategies reported to date vary widely. Antibiotic regimens differ in drug selection, duration, and combinations across studies. Probiotic interventions also differ in strains, routes of administration (vaginal vs. oral), dosages, and treatment durations. As a result, it remains difficult to attribute observed reproductive benefits to any specific component of the intervention. Accordingly, the current evidence should be interpreted primarily at the strategy level rather than as definitive proof of efficacy for a specific drug or probiotic formulation. In particular, the most consistent signal across clinical reports appears to support a broader approach of screening followed by targeted intervention in selected patients, while acknowledging substantial uncertainty regarding optimal thresholds, candidate taxa, and standardized treatment protocols.

Collectively, uncertainty in LDM definitions, variability in transcervical sampling strategies, and susceptibility to contamination in this low‐biomass environment—together with the observational nature of most studies and the marked heterogeneity of antibiotic and probiotic interventions—remain major barriers to both causal inference and clinical translation of endometrial microbiome testing. Future studies should prioritize standardized sampling protocols with comprehensive contamination controls and transparent reporting, develop consensus definitions of eubiosis grounded in reproductive outcomes, and adopt pragmatic designs (e.g., protocolized screening‐and‐treatment pathways in real‐world cohorts) complemented by causal‐inference frameworks and outcome‐based threshold optimization. Until such standardization and validation are achieved, endometrial microbiota results should be interpreted cautiously, particularly when used to guide clinical decision‐making in infertility and recurrent implantation failure.

## 5 Conclusions

Although the study of the endometrial microbiota spans only about a decade, it is now considered an important component of the female reproductive tract, comparable to the gut microbiota. Its role in implantation has been suggested, and an increasing number of studies are exploring the relationship between the endometrial microbiota and pregnancy, particularly through the use of 16S rRNA‐based microbiome testing and treatment using vaginal *Lactobacillus* suppositories. However, solid evidence is lacking. Precisely because conducting RCTs is challenging in this field, it is essential to evaluate outcomes in real‐world clinical practice carefully under standardized, protocolized testing‐and‐treatment pathways, share findings among fertility specialists, and work toward a clearer understanding of this emerging field.

## Funding

This research was supported by JSPS (Grants JP23K15827, JP24K23524, JP23K27176, JP23K24481, JP24K22157, JP23K23803, and JP24K21911), AMED (Grants JP24gn0110085, JP24gn0110069, JP24gk0210039, and JP24lk0310083), JST (Grant JPMJFR210H), and Children and Families Agency (Grant JPMH23DB0101).

## Conflicts of Interest

The authors declare no conflicts of interest.

## Data Availability

Data sharing not applicable to this article as no datasets were generated or analyzed during the current study.
